# Massive splenic calcification in amyloidosis with normal labs and physical exam

**DOI:** 10.11604/pamj.2019.32.136.18526

**Published:** 2019-03-22

**Authors:** Gaurav Dhansukhlal Gheewala, Ifeoma Oriaku

**Affiliations:** 1 Department of Anesthesiology and Critical Care, DeBakey Heart and Vascular Center, Houston Methodist Hospital, Houston, Texas, USA; 2 Department of Medicine, Houston Methodist Hospital, Houston, Texas, USA

**Keywords:** AL amyloidosis, primary amyloidosis, isolated splenic calcification

## Image in medicine

A 64-years-old male with medical history of hypertension, type II diabetes, coronary artery disease, and AL amyloidosis involving the heart, kidney and liver was admitted for decompensated heart failure, requiring Intra-Aortic Balloon Pump (IABP) and inotropic support. The patient was listed for heart transplant, but later developed cardiac arrest and required Veno-Arterial Extracorporeal Membrane Oxygenation (VAECMO) treatment. Abdominal X-ray was obtained to confirm the position of feeding tube which showed massive intra-peritoneal calcification (A). CT abdomen was obtained, and he was found to have extensive splenic calcification measures 8.9 x 7.1cm in cross-section and it spans 8.9cm in craniocaudal direction (B). Spleen was impalpable on physical exam. Before the cardiac arrest, patient's renal function was at baseline, with the remaining of the basic metabolic panel normal. Hepatic function panel was significant for mild elevation in alkaline phosphatase and alanine aminotransaminases. All other laboratory work including coagulation and complete blood count studies were normal. Nonetheless, patient's condition deteriorated over the course and he expired. Amyloid fibrils have an affinity to calcium and can result in calcific deposition in organs. Literature review showed that in amyloidosis, splenic calcification occurs in conjunction with other organs calcification especially liver. Thus this patient's case highlights the unique presentation of isolated massive splenic calcification without any other organ calcification with relatively normal laboratory and physical examination except findings consistent with his heart failure diagnosis.

**Figure 1:**
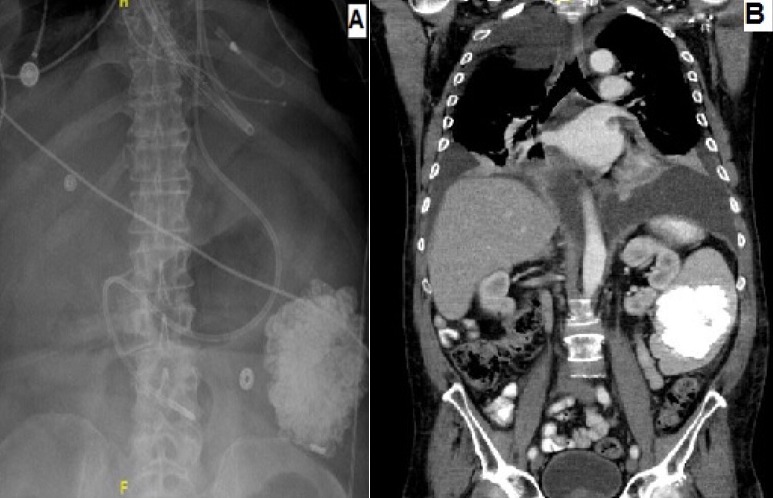
A) abdominal X-ray showing massive intra-peritoneal calcification; B) CT abdomen, showing extensive splenic calcification

